# Prehospital pulse oximetry: a red flag for early detection of silent hypoxemia in COVID-19 patients

**DOI:** 10.1186/s13054-020-03036-9

**Published:** 2020-06-08

**Authors:** Romain Jouffroy, Daniel Jost, Bertrand Prunet

**Affiliations:** Paris Fire Brigade, Emergency Medicine Department, 1 Place Jules Renard, 75017 Paris, France

The COVID-19 pandemic, which has been expanding since the first cases in Asia in late 2019, may result in acute respiratory failure (ARF) with severe hypoxemia [1–3]. In prehospital settings, the paucity of clinical respiratory signs has made assessing the severity of some COVID-19 patients challenging. Indeed, even though hypoxic ARF generally leads to an increase in respiratory rate (RR) [4], in some COVID-19 patients, a persistent normal RR was inconsistent with the severity of hypoxia.

Based on retrospective data, we aimed to describe the discrepancy between prehospital initial RR (RRi) and initial SpO2 (Spo2i; i.e., before oxygen supplementation, FiO2 = 21%) in COVID-19 patients suffering from ARF.

We retrospectively examined consecutive COVID-19 patients suffering from ARF who were treated by the Paris Fire Brigade’s basic life-support (BLS) teams in the prehospital setting. Data were provided from primary home care providers. Based on a previous study [5], we used the SpO2i/RRi ratio as an estimator of the discrepancy insofar as a low numerator is associated with hypoxia, whereas a high denominator is typically associated with respiratory failure.

After having measured the SpO2i/RRi values in COVID-19 patients, we compared them to those of non-COVID-19 patients (i.e., patients with other causes of ARF treated by the BLS teams over the previous 3 years in the same period).

Continuous data were described as median (interquartile range) and were compared by applying the Kruskal-Wallis test. The French Society of Anaesthesia and Intensive Care approved the trial protocol on April 7, 2020 (IRB 00010254-2020-055).

The study examined 1201 patients who experienced COVID-19 between March 13 and 29, 2020. The median SpO2i/RRi value was significantly higher than that of patients treated in the previous 3 years (5 [4, 5] in 2020 versus 3.4 [2.4–4.5] in 2019, 3.3[2.2–4.4] in 2018, and 3.5[2.5–4.6] in 2017, *p* < 0.001, Fig. 1).

**Fig. 1:**
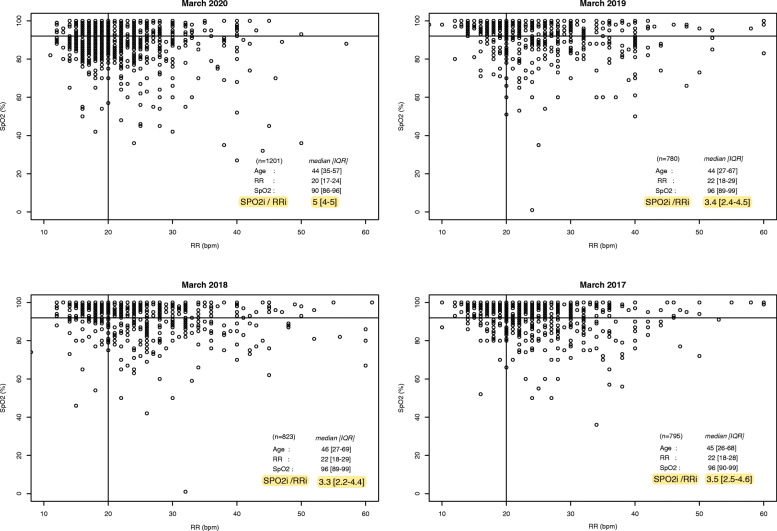
Scatter plot representing the initial SPO2 (SPO2i) and initial respiration rate (RRi) values for each patient, for COVID-19 (March 2020) and non-COVID-19 patients from the previous 3 years. The horizontal and vertical lines indicate the threshold values of SPO2 95% and respiration rate, 20 breaths per minute, respectively. We used the Kruskal-Wallis test to compare the median SPO2i/RRi value, between the period “13^th^ to March 29, 2020,” and the three previous years (*p* value < 0.001). RRi, initial respiratory rate; SpO2i, initial pulse oximetry value; *N*, number of patients included

In summary, this retrospective study based on prehospital first responder data highlighted a relatively higher discrepancy between SpO2i and RRi in COVID-19 ARF patients, in comparison with previous non-COVID-19 ARF patients. Without a systematic SpO2i measurement, a normal breathing rate could mask profound hypoxia and make severity assessment in COVID-19 patients all the more difficult in an out-of-hospital setting.

Despite differences in worldwide prehospital emergency medical services, pulse oximetry is an accessible tool that prehospital healthcare providers can easily use.

In conclusion, prehospital pulse oximetry might be used as a red flag for early detection of “silent hypoxemia” in COVID-19 patients. The prehospital SpO2i/RRi ratio needs further investigation because it might help to identify non-clinically obvious ARFs.

## Data Availability

The datasets used and/or analyzed during the current study are available from the corresponding author on reasonable request.
